# Vascular Tone Regulation Induced by C-Type Natriuretic Peptide: Differences in Endothelium-Dependent and -Independent Mechanisms Involved in Normotensive and Spontaneously Hypertensive Rats

**DOI:** 10.1371/journal.pone.0167817

**Published:** 2016-12-09

**Authors:** Carolina Caniffi, Flavia M. Cerniello, María N. Gobetto, María L. Sueiro, María A. Costa, Cristina Arranz

**Affiliations:** Universidad de Buenos Aires, Facultad de Farmacia y Bioquímica, Cátedra de Fisiología, CONICET, Instituto de Química y Metabolismo del Fármaco (IQUIMEFA), Buenos Aires, Argentina; University of Southampton, UNITED KINGDOM

## Abstract

Given that the role of C-type natriuretic peptide (CNP) in the regulation of vascular tone in hypertensive states is unclear, we hypothesized that impaired response of the nitric oxide system to CNP in spontaneously hypertensive rats (SHR) could affect vascular relaxation induced by the peptide in this model of hypertension, and that other endothelial systems or potassium channels opening could also be involved. We examined the effect of CNP on isolated SHR aortas, and the hindlimb vascular resistance (HVR) in response to CNP administration compared to normotensive rats. Aortas were mounted in an isometric organ bath and contracted with phenylephrine. CNP relaxed arteries in a concentration-dependent manner but was less potent in inducing relaxation in SHR. The action of CNP was diminished by removal of the endothelium, inhibition of nitric oxide synthase by N^ω^-nitro-L-arginine methyl ester, and inhibition of soluble guanylyl cyclase by 1H-[1,2,4]oxadiazolo[4,3-alpha]quinoxalin-1-one in both groups. In contrast, blockade of cyclooxygenase or subtype 2 bradykinin receptor increased CNP potency only in SHR. In both Wistar and SHR, CNP relaxation was blunted by tetraethylammonium and partially inhibited by BaCl_2_ and iberiotoxin, indicating that it was due to opening of the Kir and BKCa channels. However, SHR seem to be more sensitive to Kir channel blockade and less sensitive to BKCa channel blockade than normotensive rats. In addition, CNP decreases HVR in Wistar and SHR, but the effect of CNP increasing blood flow was more marked in SHR. We conclude that CNP induces aorta relaxation by activation of the nitric oxide system and opening of potassium channels, but the response to the peptide is impaired in conductance vessel of hypertensive rats.

## Introduction

C-type natriuretic peptide (CNP) is extensively distributed in the cardiovascular system, particularly in vascular endothelial cells [1,2]. Given that CNP is an important vasodilator with few renal actions, it has been suggested that this peptide has a function as a paracrine/autocrine mediator to regulate vascular smooth muscle tone and blood flow [1,3]. Physiological effects of CNP are mainly mediated through the membrane-integrated natriuretic peptide receptors subtypes B and C (NPR-B and NPR-C, respectively), which are strongly expressed in venous tissue, aortic smooth muscle and aortic endothelial cells [4,5].We previously demonstrated that acute CNP administration decreases mean arterial pressure and increases excretion of nitric oxide (NO) metabolic end products in hypertensive rats [6]. In addition, in our previous studies we showed that the peptide also increases endothelial NO synthase (eNOS) activity through NPR-C-coupled Gi protein activation in aorta of spontaneously hypertensive rats (SHR). The response of the NO system to CNP is lower in hypertensive than in normotensive rats [6,7]. It is well documented that endothelial production of NO causes vasorelaxation primarily by activating soluble guanylyl cyclase (sGC) in smooth muscle cells and by increasing intracellular cGMP, which in turn activates protein kinase G to induce vasorelaxation by decreasing cytosolic Ca^2+^ concentration [8,9].

On the other hand, it has been suggested that CNP induces hyperpolarization of microvascular endothelial cells, activating Ca^2+^-dependent K^+^ channels and involving the NPR-B receptor, protein kinase G, eNOS and sGC [10]. Other authors have postulated that CNP acts as an endothelium-derived hyperpolarizing factor via NPR-C in coronary and mesenteric resistance vessels [11,12].

The SHR is a model of hypertension with enhanced vascular tone. As in human hypertension, SHR present endothelial dysfunction with a decrease in the bioavailability and effectiveness of NO [13]. Aortas of SHR also exhibit enhanced production of reactive oxygen species (ROS), activation of endothelial cyclooxygenase-1 (COX-1), release of endothelium-derived contracting factors and prostacyclin (PGI_2_), which causes little or no relaxation in the aorta of SHR because expression of the PGI_2_ receptor is systematically lower than in normotensive rats, and PGI_2_ could also activate thromboxane prostanoid receptors [14–17]. However, in response to other stimuli, production of thromboxane A_2_ can also contribute to enhanced vascular tone in SHR [18].

The vasoactive nonapeptide bradykinin (BK), which is the main effect or of the kallikrein-kinin system, can be generated both systemically and locally within the vascular wall in both endothelium and smooth muscle cells [19]. BK is one of the most potent stimulators of NO and PGI_2_ release by endothelial cells [19,20]. In SHR, enhanced BK degradation may contribute to the endothelial dysfunction observed in these animals [21], even though young hypertensive rats seem not to present lower levels of BK in tissues and, on the contrary, present elevated levels of BK, as well as BK metabolites, as observed by Campbell and coworkers [22].

Considering this background and the fact that the role of CNP in the regulation of vascular tone in hypertensive states is unclear, we hypothesized that the impaired response of the NO system to CNP in SHR could affect vascular relaxation induced by the peptide in this model of hypertension but other endothelial systems or potassium channels could also contribute to this impaired response. The capability of central vessels to respond to cardiovascular performance modifies arterial blood pressure and flow dynamics [23]. In addition to structural changes, arterial stiffness is strongly affected by endothelial function and vascular smooth muscle tone [23,24]. Therefore, we investigated the mechanisms involved in the vasodilator effect of CNP in a genetic model of hypertension by studying endothelium derived NO, involvement of the prostanoid and kinin systems and participation of potassium channels.

## Materials and Methods

### Animals

Fourteen-week-old male Wistar and SHR rats were obtained from the breeding laboratory of Facultad de Farmacia y Bioquímica and Instituto de Investigaciones Médicas A. Lanari, Facultad de Medicina (Universidad de Buenos Aires, Argentina). Rats were housed in a humidity- and temperature-controlled environment with an automatic 12-hour light/dark cycle. They were fed standard rat chow from Nutrimentos Purina (Buenos Aires, Argentina) and tap water ad libitum up to the day of the experiments. Systolic blood pressure (SBP) was measured in awake animals (tail cuff method) with a MP100 Pulse Transducer, PanLab (Quad Bridge Amp, ADInstruments), and recorded with a polygraph (Quad Bridge Amp, ADInstruments). Data were obtained using data acquisition software (PowerLab 8/30 and Labchart, Australia).

### Experimental design

All experimental protocols were performed in accordance with the Guide for the Care and Use of Laboratory Animals (National Institutes of Health, Publication No. 85–23, Revised 1996) and Regulation No. 6344/96 of Argentina’s National Drug, Food and Medical Technology Administration (ANMAT). Experimental procedures were approved by the Ethics Committee of the School of Pharmacy and Biochemistry (CEFFB), Universidad de Buenos Aires, Argentina.

### Preparation of isolated aortic rings

Animals were randomly assigned to each protocol. The thoracic aorta from Wistar and SHR was removed immediately after decapitation and placed in cold oxygenated Krebs buffer. The aorta was carefully cleaned of fat and connective tissue and then cut into 3-5-mm long ring segments. Endothelium was denuded in some rings by gently rubbing the lumen of aortic segments. Rings were mounted in 10 mL organ baths filled with oxygenated Krebs buffer at 37 ± 0.5°C (95% O_2_, 5% CO_2_). The composition of the Krebs buffer was (mmol/L): 118 NaCl; 4.7 KCl; 25 NaHCO_3_; 1.13 NaH_2_PO_4_; 2.55 CaCl_2_; 1.15 MgCl_2_; 11.1 D-glucose; 0.004 EDTA; 0.11 ascorbic acid, pH 7.4.

Isometric tension (g) was measured using a force displacement transducer connected to a PowerLab with a LabChart Software recording system (AD Instruments, Australia). Aortic rings were then progressively stretched to an optimal basal tension of 1 g and allowed to equilibrate for 60 minutes. During this period, the bathing solution was replaced every 15 minutes and, if needed, the basal tone was readjusted to 1 g. After control stimulation with KCl (90 mmol/L) and a 30-minute recuperation period, acetylcholine (10 μmol/L) was added to phenylephrine (10 μmol/L) precontracted vessels to verify endothelial integrity. Endothelium-intact rings were discarded when relaxation with acetylcholine was less than 80%. The composition of the Krebs buffer enriched with K^+^ was (mmol/L): 32.8 NaCl; 90 KCl; 25 NaHCO_3_; 1.13 NaH_2_PO_4_; 2.55 CaCl_2_; 1.15 MgCl_2_; 11.1 D-glucose; 0.004 EDTA; 0.11 ascorbic acid, pH 7.4. After the equilibration period, specific protocols were performed on rat aortic rings.

### Endothelium-dependent effect of CNP on vascular reactivity

Cumulative concentration-response curves were constructed to CNP (10 pmol/L to 1 μmol/L) or cANP_(4–23)_ (NPR-C selective agonist, 1 fmol/L to 1 nmol/L) in phenylephrine-precontracted rings (at EC80 of the maximum response to avoid differences between treated and untreated aortic rings, S1 Fig) in the presence or absence of: N^ω^-L-arginine methyl ester (L-NAME, non-selective NOS inhibitor, 0.1 mmol/L); 1H-[1,2,4]Oxadiazolo[4,3-a]quinoxalin-1-one (ODQ, selective sGC inhibitor, 1 μmol/L); indomethacin (Indo, non-selective COX inhibitor, 10 μmol/L); D-Arg[Hyp3,Thi5,D-Tic7,Oic8]-bradykinin (HOE 140, bradykinin subtype 2 (B_2_) receptor antagonist, 0.1 mmol/L). Each blocker was administered 30 minutes before the addition of CNP. Concentrations of CNP and cANP_(4–23)_ used are different since the maximal response was achieved with lower concentration of NPR-C selective agonist. Only one curve to any one blocker was constructed in any single tissue. NO-mediated relaxation was determined by measuring the portion of CNP-induced relaxation that was abolished by L-NAME. Calculations were performed by determination of the area under the curve (AUC, in arbitrary units, au) of individual dose-response curves. The NO-mediated response was then calculated as the difference between the AUC of CNP-induced relaxation in the absence and presence of L-NAME. In order to evaluate the capability of smooth muscle to respond to NO in both groups, additional experiments using sodium nitroprusside (SNP, NO donor, 0.1 pmol/L to 1 μmol/L) were performed. All drugs were purchased from Sigma-Aldrich.

### Endothelium-independent effect of CNP on vascular reactivity

The tests were conducted in endothelium-denuded aortic rings to evaluate the possible role of potassium channels from smooth muscle in the relaxant effect of CNP. To show that removal of the endothelium was successful, failure of acetylcholine (10 μmol/L) to relax the rings precontracted with phenylephrine (10 μmol/L) was used. In endothelium-denuded rings, cumulative concentration-response curves were constructed to CNP (10 pmol/L to 1 μmol/L) in phenylephrine-precontracted rings at EC80, in the presence or absence of: tetraethylammonium (TEA, non-selectively potassium channel blocker, 1 mmol/L); BaCl_2_ (inwardly rectifying potassium channel (Kir) blocker, 30 μmol/L); glibenclamide (Glib, ATP-dependent potassium channel blocker, 3 μmol/L); iberiotoxin (IbTx, selective large-conductance calcium-activated potassium channel (BKCa) blocker, 0.1 μmol/L); either alone or in combination. Each blocker was administered 30 minutes before the addition of CNP. Only one curve to any one blocker was constructed in any single tissue.

### Determination of NOS activity and Western blot analysis

NOS activity was measured in aorta rings treated with CNP at a concentration that produces 50% of the maximal response in rings precontracted with phenylephrine. NOS activity was measured using [^14^C] L-arginine as substrate, as previously described [6,7]. Aortic rings from Wistar and SHR rats with or without endothelium were subjected to the same procedure described above except that, after the maximum vasoconstrictor response to phenylephrine was reached, CNP was added at a concentration that produces 50% of the maximal vasorelaxant response. After maximal effect of CNP, rings were removed and placed in stop buffer containing 0.5 mmol/L EGTA, 0.5 mmol/L EDTA and 20 mmol/L HEPES (pH 5.5). Tissue samples were then homogenized in the stop solution and the homogenates were centrifuged at 12,000 g for 20 minutes and separated by Dowex AG 50W-X8 columns (Na^+^ form, Bio-Rad). The amount of [^14^C] L-citrulline was determined with a liquid scintillation counter (Wallac 1414 WinSpectral). Specific NOS activity was assessed in the presence of L-NAME (0.1 mmol/L). Western blotting was performed in samples of aorta containing equal amounts of protein (0.080 mg protein/lane) and separated by electrophoresis in 7.5% SDS-polyacrylamide gels, transferred to a nitrocellulose membrane (Bio-Rad, Munich, Germany), and then incubated with rabbit polyclonal anti-eNOS antibody (1/500 dilution, epitope at the NH2 terminus, Santa Cruz Biotechnology, CA, USA) or rabbit anti-phospho-eNOS (Ser^1177^, 1/500 dilution, Cell Signaling Technology Inc, Danvers, MA, USA) and a horseradish peroxidase-conjugated goat anti-rabbit secondary antibody (1/5,000 dilution). An anti-β-actin antibody (1/5,000 dilution, Sigma Aldrich Chemical Co, St Louis, MO) was used as a loading control and data were normalized to β-actin expression [6]. Samples were revealed by chemiluminescence using an enhanced chemiluminescence reagent (Amersham Pharmacia Biotechnology, Uppsala, Sweden) for 2–4 minutes. Quantification of the bands was performed by digital image analysis using a Hewlett-Packard scanner and Image J software (NIH—National Institute of Health, USA). All experiments were performed by triplicate [6].

### Hind limb vascular resistance measurement

In order to evaluate if the effect of CNP on resistance vessels differs between normotensive and hypertensive rats, we calculated hindlimb vascular resistance (HVR) from the mean arterial pressure (MAP) divided by hindlimb blood flow (HBF) before and after peptide infusions. The infusions were performed by triplicate with each peptide and when stable HBF values were achieved, the mean was calculated.

Rats were anesthetized with (urethane, 1 g/kg i.p., Sigma Aldrich, USA) [6,7] and a tracheotomy was performed to allow spontaneous breathing. Body temperature was maintained at 37 ± 0.5°C using a heating pad controlled by rectal temperature. Blood pressure (BP) and heart rate were measured by a SP 844 transducer (MEMScAP AS, Norway) connected to a polyethylene cannula (PE-50, A-M systems, Carlsborg, USA) filled with heparinized saline (50 IU/mL) and inserted into the left carotid artery. HBF was continuously measured by a transit-time ultrasound flow probe (model 1PRB3970) and flowmeter (model TS420 Perivascular Flowmeter Module, Transonic Systems Inc., NY, USA) placed around the abdominal aorta 1–2 mm proximal to the iliac bifurcation. Innocuous water-soluble ultrasound gel was applied over the opened area to keep tissue hydrated and to maintain adequate flow signal. A polyethylene cannula (PE-10, Laboratorios Rivero, Argentina) was inserted in the right iliac artery and advanced to the bifurcation of aorta to allow for direct arterial injection into the left hindlimb circulation. All hemodynamic variables along with HBF were digitally displayed and recorded by Power Lab with a LabChart Software (AD Instruments, Australia).

After 10 minutes of stabilization period with saline infusion at 50 μL/min, the vasodilator responses to CNP (1 μmol/L) and cANP_(4–23)_ (10 nmol/L) were evaluated by bolus administration directly into the left hindlimb circulation. The maximal doses of these peptides without changes in blood pressure or heart rate were used. The stock solutions of these agents were adjusted such that the volume injected was constant at 100 μL, which was injected over 10 s followed by prompt resumption of saline infusion at 50 μL/min. In order to verify the vasodilator response, acetylcholine (3 nmol/kg and 0.3 nmol/kg) was administrated. The doses infused were kept below the level at which changes of MAP or heart rate may occur, thus ensuring no or minimal overflow into general circulation at the concentrations used [25].

### Statistical Analysis

All values are expressed as mean ± SEM and, each set of experiments were performed by triplicate per rat and n indicates the number of rats used. The Prism program (Graph Pad Software, Inc., San Diego, CA, USA) was used for statistical analysis. The values of vascular reactivity responses to CNP are expressed as a percentage of the preceding contraction induced by phenylephrine. The concentration of the agonist producing a half-maximal response (EC_50_) was determined after *logit* transformation of the normalized concentration-response curves, and it is reported as the negative logarithm (pEC_50_) of the mean of individual values for each tissue. The maximal relaxant effect (Emax) was considered to be the maximal amplitude response reached in concentration-effect curves to CNP.

The results of each variable for each experimental group were analyzed with a two-way analysis of variance (ANOVA), where one factor was the different treatments and the other the genotypes (Wistar or SHR). The effect of each factor was tested independently of the effect of the other, as well as the interaction between both factors. When no interaction between the two factors was found, the Bonferroni post hoc test for multiple comparisons of the main effects was applied. In cases where an interaction between the two factors was found, the interaction was reported and the simple effects were analyzed using the Bonferroni post hoc test for multiple comparisons between the subgroups of interest. Comparisons between two groups were analyzed by unpaired Student *t* test. *P* value < 0.05 was considered a significant difference.

## Results

At 14-week old, SHR displayed higher SBP values vs. Wistar rats (Wistar = 119 ± 2; SHR = 189 ± 3*; * p < 0.001 vs. Wistar).

### Endothelium-dependent effect of CNP on vascular reactivity

**Fig 1:**
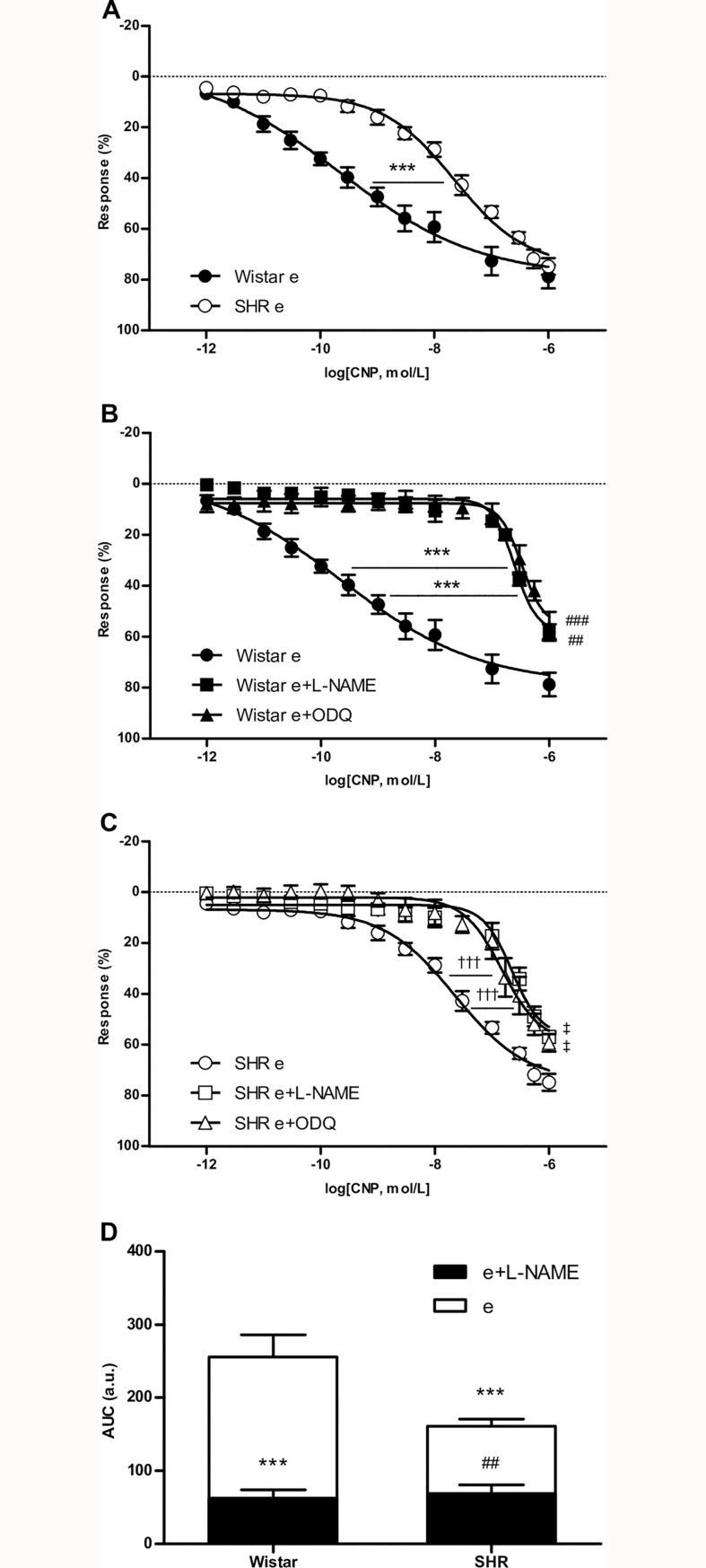
Participation of NO pathway in CNP induced relaxation. Emax: maximal relaxant effect; pEC_50_: negative logarithm of the concentration of the agonist producing a half-maximal response; AUC: area under the curve. Results are expressed as means ± SEM, n = 10 rats / group. (A) Relaxation curves of aortic arteries with an intact endothelium (e) from Wistar or SHR; ***p< 0.001 for SHR e vs. Wistar e pEC_50_ values. (B) Wistar concentration-response curves in the absence or presence of L-NAME (0.1 mmol/L) or ODQ (1 μmol/L); ***p < 0.001 vs. Wistar e pEC_50_ values; ^##^p< 0.01 Wistar e+L-NAME vs. Wistar e Emax values; ^###^p< 0.001 Wistar e+ODQ vs. Wistar e Emax values. (C) SHR concentration-response curves in the absence or presence of L-NAME (0.1 mmol/L) or ODQ (1 μmol/L); ^†††^p < 0.001 vs. SHR e pEC_50_ values; ^‡^p < 0.05 vs. SHR e Emax values. (D) AUC based on the individual concentration-dependent relaxation curves to CNP; ***p < 0.001 vs. Wistar NO-dependent response; ^##^p < 0.01 vs. SHR NO-dependent response.

The cumulative addition of CNP to the organ bath solution during sustained contraction induced by phenylephrine was able to promote concentration-dependent relaxation with similar maximum effect in intact endothelium aortic rings from Wistar and SHR (Fig 1A). However, the AUC of vascular response to CNP in SHR was 62.9% of the response in Wistar rats (Fig 1D) and the relaxation induced by CNP was less potent in aortic rings from SHR than in aortic rings from Wistar rats (Table 1).

**Table 1:** Endothelium-dependent effect of CNP on vascular reactivity.

			Emax (%)	pEC_50_
**Wistar**	**CNP**	e	79.8 ± 4.6	9.64 ± 0.27
		e + L-NAME	59.1 ± 2.9**	6.61 ± 0.04***
		e + ODQ	57.0 ± 5.7***	6.49 ± 0.05***
		e + Indo	75.4 ± 5.0	9.36 ± 0.30
		e + HOE 140	71.8 ± 9.7	9.19 ± 0.53
		ne	54.8 ± 7.3**	6.74 ± 0.10***
	**cANP**_**(4–23)**_	e	36.7 ± 2.2***	12.51 ± 0.36***
**SHR**	**CNP**	e	74.8 ± 3.3	7.64 ± 0.15***
		e + L-NAME	56.9 ± 2.0^†^	6.65 ± 0.05^†††^
		e + ODQ	58.3 ± 3.5^†^	6.82 ± 0.06^†††^
		e + Indo	75.2 ± 1.2	9.45 ± 0.49^††^
		e + HOE 140	66.4 ± 6.3	9.50 ± 0.48^††^
		ne	55.6 ± 3.1^†^	6.78 ± 0.06^††^
	**cANP**_**(4–23)**_	e	25.5 ± 2.2***	11.66 ± 0.19^†††^^◊◊◊^

L-NAME: non-selective NOS inhibitor (0.1 mmol/L); ODQ: sGC inhibitor (1 μmol/L); Indo: indomethacin, non-selective COX inhibitor (10 μmol/L); HOE 140: B_2_ antagonist (0.1 mmol/L); cANP_(4–23)_: NPR-C selective agonist; e: intact endothelium; ne: without endothelium. Emax: maximal relaxant effect; pEC_50_: negative logarithm of the concentration of the agonist producing a half-maximal response. Results are expressed as means ± SEM.

**p < 0.01 vs. Wistar CNP e

***p < 0.001 vs. Wistar CNP e

^†^p < 0.05 vs. SHR CNP e

^††^p < 0.01 vs. SHR CNP e

^†††^p < 0.001 vs. SHR CNP e

^◊◊◊^p < 0.001 vs. Wistar cANP_(4–23)_ e.

NOS blockade with L-NAME reduced the maximal relaxation induced by CNP in aortic rings from Wistar and SHR (Table 1), and pEC_50_ was lower in both groups when compared with CNP relaxation in the absence of L-NAME (Fig 1B and 1C). Similar results were obtained with sGC inhibitor in both Wistar and SHR, suggesting that CNP-induced relaxation is partially mediated by the NO-cGMP pathway in intact endothelium rings of both groups (Fig 1B and 1C). However, the extent of CNP-induced relaxation dependent of NO was smaller in SHR than in normotensive rats (Fig 1D).

**Fig 2:**
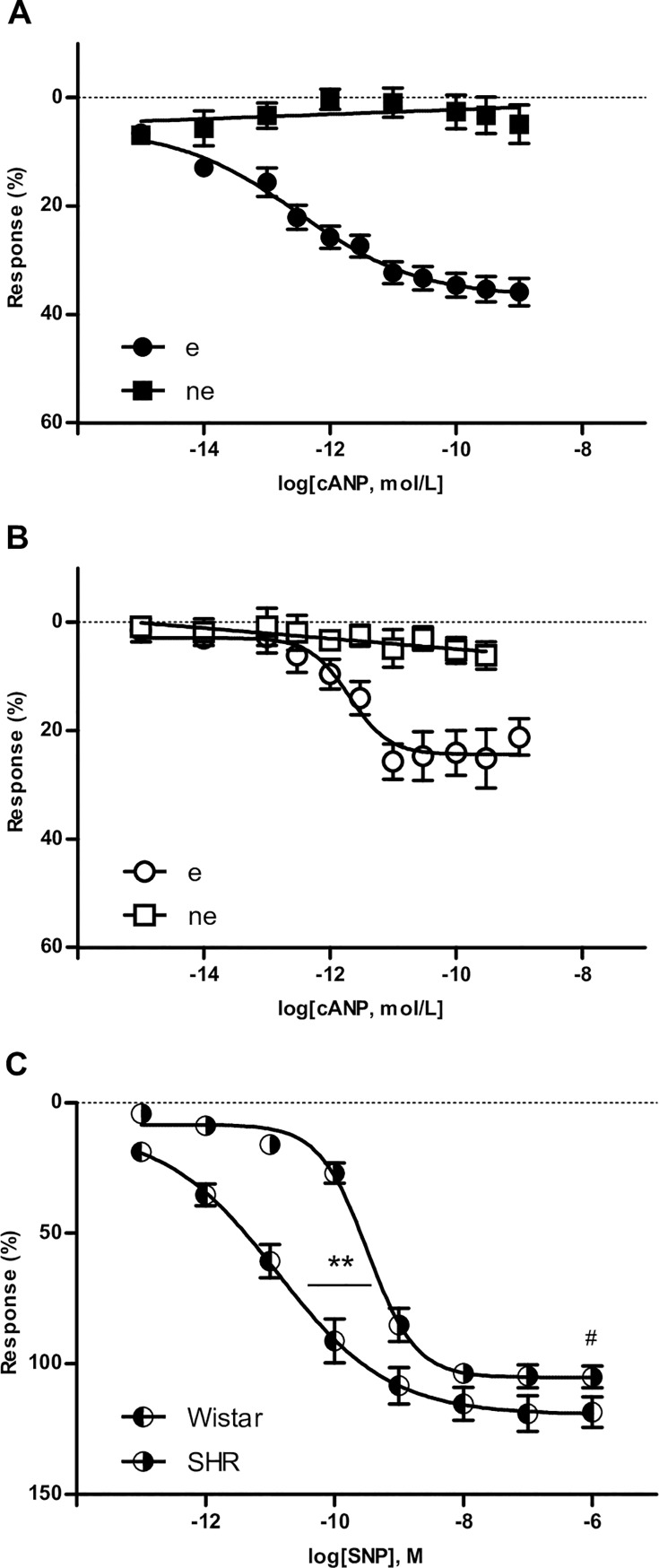
Participation of NPR-C pathway and smooth muscle response to NO in aorta relaxation. Emax: maximal relaxant effect; pEC_50_: negative logarithm of the concentration of the agonist producing a half-maximal response; cANP_(4–23)_: NPR-C selective agonist; SNP: sodium nitroprusside. Results are expressed as means ± SEM, n = 6 rats / group in A and B; n = 4 rats / group in C. (A) Wistar concentration-response curves in the absence (ne) or presence (e) of an intact endothelium; (B) SHR concentration-response curves in the absence (ne) or presence (e) of an intact endothelium. (C) Wistar and SHR concentration-response curves to SNP; **p < 0.01 vs. Wistar pEC_50_ value; ^#^p < 0.05 vs. Wistar Emax value.

In addition, relaxation induced by NPR-C activation was lower compared to CNP induced response and, the absence of an intact endothelium, abolished the vasodilator effect of NPR-C stimulation in both groups (Fig 2A and 2B). In order to determine if the difference observed in NO-dependent response between both groups is due to an altered capacity of smooth muscle to response to this mediator, cumulative concentration-response curves were constructed to SNP (Fig 2C). Aortas from SHR showed lower Emax (Wistar = 119.5 ± 4.4 vs. SHR = 105.3 ± 2.3; p < 0.05) and pEC_50_ (Wistar = 10.89 ± 0.29 vs. SHR = 9.50 ± 0.07; p < 0.01) compared to normotensive rats.

**Fig 3:**
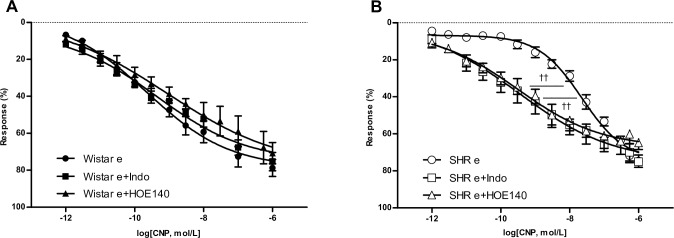
Effects of COX inhibition or B_2_ antagonism on the relaxation induced by CNP. pEC_50_: negative logarithm of the concentration of the agonist producing a half-maximal response; e: intact endothelium. Results are expressed as means ± SEM, n = 6 rats / group. (A) Wistar concentration-response curves in the absence or presence of Indo (10 μmol/L) or HOE 140 (0.1 mmol/L); ns. (B) SHR concentration-response curves in the absence or presence of Indo (10 μmol/L) or HOE 140 (0.1 mmol/L); ^††^p < 0.01 vs. SHR e pEC_50_ values.

Aortic rings were pretreated with indomethacin in order to evaluate other endothelium-derived factors. COX inhibition with indomethacin induced no changes in the effect of CNP on vascular tone in normotensive rats (Fig 3A). However, in SHR aortic rings, indomethacin preincubation to inhibit PG synthesis induced an increase in pEC_50_ of CNP with no changes in the maximal relaxant response of the peptide (Fig 3B), indicating that PG synthesis inhibition promotes a more effective relaxant effect of CNP in hypertensive rats versus normotensive rats.

Similar results were obtained with HOE 140, a B_2_ receptor antagonist (Table 1). In aortic rings from SHR, CNP was able to induce a more potent relaxant effect in the presence of B_2_ receptor blocker, but HOE 140 did not modify the curve obtained with CNP in Wistar aortic rings as a discernible difference (Fig 3A and 3B).

On the other hand, in endothelium-denuded rings, endothelium removal diminished the maximal vasodilator response to CNP and pEC_50_ in both groups (S2 Fig). In fact, similar values of Emax and pEC_50_ for CNP were obtained in aortic rings with intact endothelium in the presence of L-NAME and in denuded endothelium aortic rings (Table 1).

### Determination of NOS activity and Western blot analysis

**Fig 4:**
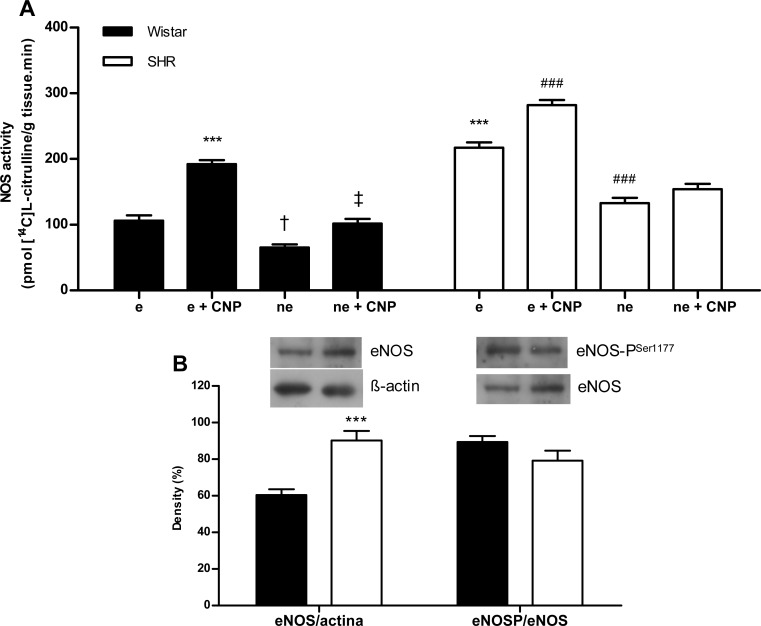
Effect of CNP on NOS activity in aortic rings with (e) and without endothelium (ne). Results are expressed as means ± SEM, n = 8 rats / group. (A) NOS activity. Statistical analysis: two-way ANOVA followed by Bonferroni post hoc test; interaction treatment x strain: p< 0.001; simple effects were analyzed by Bonferroni post hoc test; ***p< 0.001 vs. Wistar e; ^†^p< 0.05 vs. Wistar e; ^‡^p< 0.05 vs. Wistar ne; ^###^p< 0.001 vs. SHR e. (B) NOS protein expression. Statistical analysis: unpaired Student *t* test to compare between two groups; ***p< 0.001 vs. Wistar.

Fig 4A shows that the activity of NOS was higher in aortas with endothelium from SHR compared with Wistar. In addition, aortic eNOS protein content was higher in SHR than in Wistar rats, and phosphorylation level at Ser^1177^ resulted similar in both groups (Fig 4B).

Also, Fig 4A shows the effect of CNP on NOS activity in aortic rings with and without endothelium from Wistar and SHR rats. NOS activity increased significantly in both groups after CNP addition, but the response of vascular NOS to CNP was more marked in aorta from Wistar rats (Δ_[(E+CNP)-E]Wistar_ = 85.9 ± 6.4; Δ_[(E+CNP)-E]SHR_ = 54.8 ± 7.2*; * p < 0.01 vs. Δ_Wistar_). On the other hand, endothelium-denuded aortic rings presented lower NOS activity, and although CNP augmented enzyme activity similarly in both Wistar and SHR (Δ_[(NoE+CNP)-NoE]Wistar_ = 36.7 ± 7.0; Δ_[(NoE+CNP)-NoE]SHR_ = 41.4 ± 3.4; ns), such activity did not reach the levels found in rings with an intact endothelium (Fig 4A).

### Endothelium-independent effect of CNP on vascular reactivity

**Fig 5:**
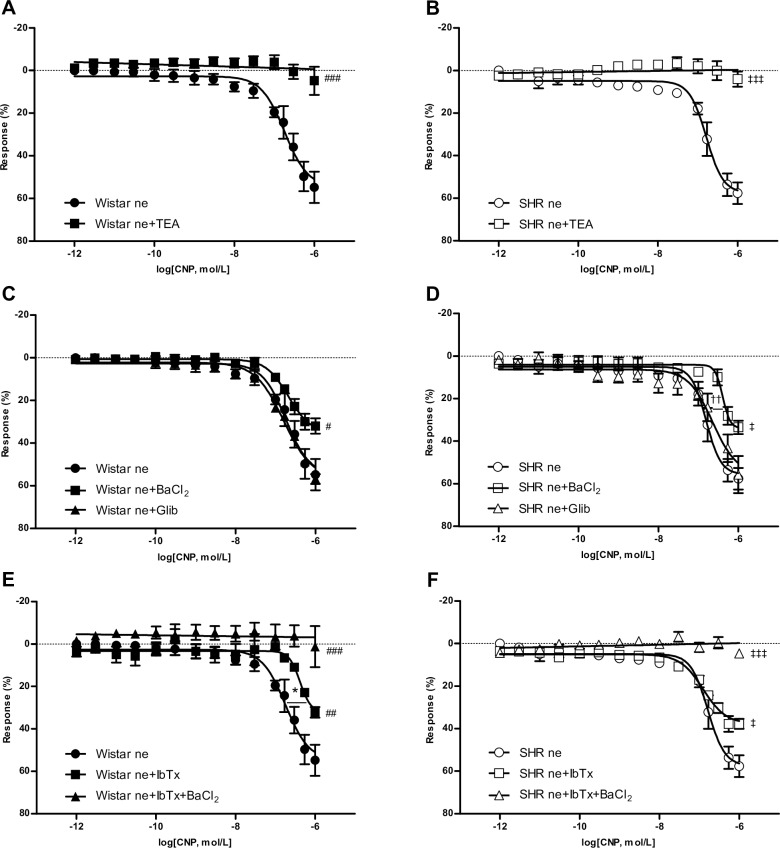
Effect of potassium channels blockade on relaxation induced by CNP. Emax: maximal relaxant effect; pEC_50_: negative logarithm of the concentration of the agonist producing a half-maximal response; ne: aortic rings without endothelium. Results are expressed as means ± SEM, n = 6 rats / group in A and B; n = 7–8 rats / group in C to F. (A) Wistar concentration-response curves in denuded aortic rings in the absence or presence of TEA (1 mmol/L); ^###^p< 0.001 vs. Wistar ne Emax values. (B) SHR concentration-response curves in denuded aortic rings in the absence or presence of TEA (1 mmol/L); ^‡‡‡^p< 0.001 vs. SHR ne Emax values. (C) Wistar concentration-response curves in denuded aortic rings in the absence or presence of BaCl_2_ (30 μmol/L) or Glib (3 μmol/L); ^#^p< 0.05 Wistar ne+BaCl_2_ vs. Wistar ne Emax values. (D) SHR concentration-response curves in denuded aortic rings in the absence or presence of BaCl_2_ (30 μmol/L) or Glib (3 μmol/L); ^††^p< 0.01 SHR ne+BaCl_2_ vs. SHR pEC_50_ values; ^‡^p< 0.05 SHR ne+BaCl_2_ vs. SHR ne Emax values. (E) Wistar concentration-response curves in denuded aortic rings in the absence or presence of IbTx (0.1 μmol/L) or IbTx+BaCl_2_ (0.1 μmol/L + 30 μmol/L); *p< 0.05 Wistar ne+IbTx vs. Wistar ne pEC_50_ values; ^##^p< 0.01 Wistar ne+IbTx vs. Wistar ne Emax values; ^###^p< 0.001 Wistar ne+IbTx+BaCl_2_ vs. Wistar ne Emax values. (F) SHR concentration-response curves in denuded aortic rings in the absence or presence of IbTx (0.1 μmol/L) or IbTx+BaCl_2_ (0.1 μmol/L + 30 μmol/L); ^‡^p< 0.05 SHR ne+IbTx vs. SHR Emax values; ^‡‡‡^p< 0.001 SHR ne+IbTx+BaCl_2_ vs. SHR ne Emax values.

In denuded endothelium aortic rings, non-selective blockade of potassium channels with TEA completely inhibited CNP-induced relaxation, suggesting the role of K^+^ channels in the effect of CNP on smooth muscle cells from both Wistar and SHR (Fig 5A and 5B).

To determine the ion channel that mediates CNP-induced hyperpolarization, the effect of pretreatment with the chemical channel blockers was evaluated. The relaxant effect of CNP on tonic contraction induced by phenylephrine was significantly reduced in the presence of the specific blocker for inwardly rectifying potassium channel, suggesting participation of the Kir channels in the relaxant effect of CNP in normotensive and hypertensive rats (Fig 5C and 5D). Moreover, although Kir channel blockade did not modify pEC_50_ in aortic rings from Wistar rats, pEC_50_ was lower in SHR rats (Table 2).

**Table 2:** Endothelium-independent effect of CNP on vascular reactivity.

		Emax (%)	pEC_50_
**Wistar**	ne	54.8 ± 7.3	6.74 ± 0.10
	ne + BaCl_2_	35.5 ± 3.6*	6.68 ± 0.11
	ne + Glib	56.8 ± 3.3	6.80 ± 0.06
	ne + IbTx	32.2 ± 3.5**	6.37 ± 0.05*
**SHR**	ne	55.6 ± 3.1*	6.78 ± 0.06**
	ne + BaCl_2_	34.0 ± 3.2^†^	6.39 ± 0.05^††^
	ne + Glib	55.7 ± 5.7	6.64 ± 0.08
	ne + IbTx	37.8 ± 3.3^†^	6.91 ± 0.05

BaCl_2_: Kir channel blocker (30 μmol/L); Glib: glibenclamide, K_ATP_ channel blocker (3 μmol/L); IbTx: iberiotoxin, BKCa channel blocker (0.1 μmol/L). Emax: maximal relaxant effect; pEC_50_: negative logarithm of the concentration of the agonist producing a half-maximal response. Results are expressed as means ± SEM.

*p < 0.05 vs. Wistar ne

**p < 0.01 vs. Wistar e

^†^p < 0.05 vs. SHR e

^††^p < 0.01 vs. SHR e.

Since an ATP-dependent member of the Kir potassium channel family (K_ATP_) has been identified [26], the effect of glibenclamide on the relaxant effect of CNP was tested. Glibenclamide failed to modify smooth muscle relaxation induced by CNP in both Wistar and SHR (Fig 5C and 5D).

On the other hand, the BKCa channel blocker IbTx attenuated the maximal response to CNP in denuded endothelium aortic rings from Wistar and SHR rats (Fig 5E and 5F). IbTx also diminished pEC_50_ in rings from normotensive rats, but no differences in pEC_50_ were observed in hypertensive rats. Finally, our results show that simultaneous addition of BaCl_2_ and IbTx completely blunted the effect of CNP on vascular smooth muscle (Fig 5E and 5F).

### Hindlimb vascular resistance evaluation

**Fig 6:**
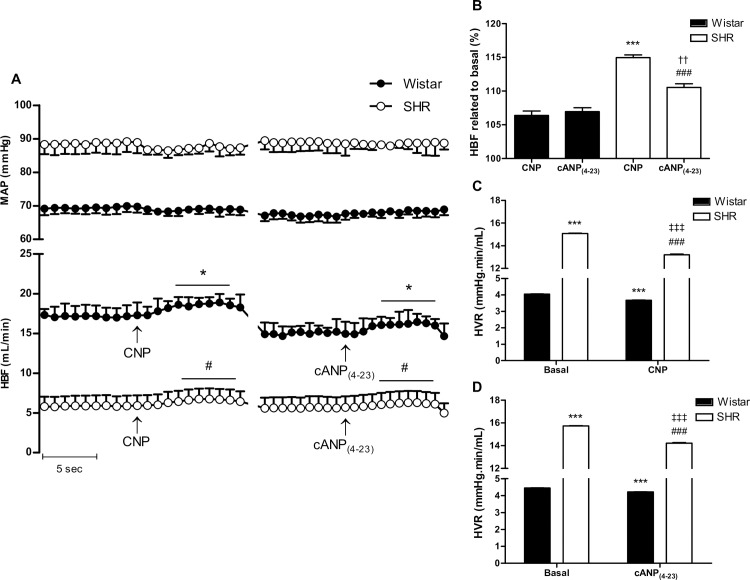
Effect of acute CNP infusion on hindlimb vascular resistance in Wistar and SHR. MAP: mean arterial pressure; HBF: hindlimb blood flow; HVR: hindlimb vascular resistance. Results are expressed as means ± SEM, n = 5 rats / group. (A) Time course data of MAP and HBF before and after intraarterial infusion of CNP (1 μmol/L) or cANP_(4–23)_ (10 nmol/L). The infusions were performed by triplicate with each peptide and when stable HBF values were achieved, the mean was calculated. The horizontal black lines show the significant differences from the corresponding baseline values. Statistical analysis: two-way ANOVA followed by Bonferroni post hoc test; interaction treatment x strain: p< 0.001; simple effects were analyzed by Bonferroni post hoc test; * p< 0.001 vs. Wistar basal; ^#^ p< 0.001 vs. SHR basal. (B) Maximal HBF response to CNP or cANP_(4–23)_ in normotensive and hypertensive rats. Statistical analysis: two-way ANOVA followed by Bonferroni post hoc test; interaction treatment x strain: p< 0.001; simple effects were analyzed by Bonferroni post hoc test; *** p< 0.001 vs. Wistar CNP; ^###^ p< 0.001 vs. SHR CNP; ^††^p< 0.01 vs. Wistar cANP_(4–23)_. (C) Comparison between basal and CNP-induced HVR. HVR was calculated from MAP divided by HBF. Statistical analysis: two-way ANOVA followed by Bonferroni post hoc test; interaction treatment x strain: p< 0.001; simple effects were analyzed by Bonferroni post hoc test; *** p< 0.001 vs. Wistar_Basal_; ^###^ p< 0.001 vs. SHR_Basal_; ^‡‡‡^p< 0.001 vs. SHR_CNP_. (D) Comparison between basal and cANP_(4–23)_-induced HVR. HVR calculated as MAP divided by HBF. Statistical analysis: two-way ANOVA followed by Bonferroni post hoc test; interaction treatment x strain: p< 0.001; simple effects were analyzed by Bonferroni post hoc test; *** p< 0.001 vs. Wistar_Basal_; ^###^ p< 0.001 vs. SHR_Basal_; ^‡‡‡^p< 0.001 vs. SHR_cANP(4–23)_.

SHR showed higher MAP and lower HBF, compared to Wistar rats (Fig 6A). The acute infusion of CNP or cANP_(4–23)_ induced an increase in HBF in both groups, without changes in MAP (Fig 6A). When we compare the percentage of change of HBF induced by CNP and cANP(4–23), we observed that the effect of cANP_(4–23)_ on HBF was similar to the effect of CNP, in Wistar rats (Fig 6B). However, lower values of HBF in response to NPR-C selective agonist were observed compared to CNP infusion in SHR when comparing HBF in response to the peptides related to basal blood flow (Fig 6B). In addition, the relative vascular response to the peptide was higher in SHR than in Wistar rats (Fig 6B). The analysis of vascular tone in resistance arteries showed that hypertensive rats have higher HVR than normotensive rats (Fig 6C and 6D). Acute infusion of CNP induced a drop in HVR in both groups (Fig 6C). Similar results were observed with the NPR-C selective agonist in both groups, but the effect was more marked in SHR (Fig 6D).

## Discussion

In the present study we demonstrated that CNP-induced relaxation of isolated aortas from normotensive and hypertensive rats involves endothelial NO production and opening of potassium channels. Differences between SHR and Wistar rats in the vascular response to CNP were also observed in conduit and resistance vessels. Elevated peripheral vascular resistance along with increased arterial stiffness are two main factors involved in development and/or maintenance of high blood pressure. In addition to structural changes, arterial stiffness is strongly affected by endothelial function and vascular smooth muscle tone [23,24]. Our results showed that CNP-induced relaxation could be one of the impaired mechanisms in hypertensive states.

Previous findings demonstrate that the role of endothelium-derived NO in CNP-induced vascular relaxation is controversial. Madhani et al. showed that endothelial NOS appears to negatively modulate the relaxant effect of CNP in mouse aortic rings, and Liang et al. showed that basally released endothelium-derived NO inhibits the response of vascular smooth muscle to the peptide [27,28]. However, Brunner and Wölkart demonstrated that NO system blockade diminishes the relaxant effect of CNP in coronary vessels [11]. In the present study we observed that relaxation induced by CNP in an intact conductance vessel requires the presence of an intact NO system and sGC activity, as well as the activation of NPR-C receptor, since CNP was less effective in inducing relaxation after NOS blockade or sGC inhibition and L-NAME blunted the response of NPR-C selective agonist in aorta. Consistently with our findings, it was recently determined that CNP-induced relaxation in intact aorta involves intracellular calcium increase and NO production in endothelial cells of normotensive rats [29].

Moreover, it is widely known that prostanoids, which are synthesized by cyclooxygenases, are also endothelium-derived factors that regulate vascular tone. Under physiological condition, resting endothelial cells synthesize and release PGI_2_ to induce vascular smooth muscle relaxation, while the release of vasoconstrictor prostanoids, such as thromboxane, is limited [30]. Therefore, we evaluated participation of prostanoids in the relaxant effect of CNP. There was no difference in the Emax to CNP in aortic rings from Wistar and SHR incubated with indomethacin. An increase in CNP relaxant potency by inhibition of prostaglandin synthesis was observed only in SHR. This result may be due to SHR having an abnormal production of prostanoids inducing activation of thromboxane prostanoid receptors of the vascular smooth muscle cells, which initiate endothelium-dependent contractions and contribute to endothelial dysfunction in this model of hypertension [30,31]. In SHR, the response of NO system to vasoactive substances is decreased, included to CNP as we demonstrated in our present work. However, we cannot dismiss the functional antagonism due to the known imbalance between vasodilator and vasoconstrictor substances in SHR. The endothelium-dependent contractions correlate with the severity of hypertension and also increase with the aging process, appearing even in normotensive aged rats [30]. However, despite these differences, the present study showed for the first time that CNP is able to induce a similar maximal relaxant response in aorta from both normotensive and hypertensive young adult rats.

Additionally, it is well established that vascular tone may also be modulated by the local kallikrein-kinin system, which is different from the circulating plasma components [32,33]. BK can modulate vascular functions via numerous mechanisms, including prostanoids, NO, intracellular calcium, potassium channels, and ROS generation [34–37]. All these events occur via two G-protein-coupled receptors: B_1_ and B_2_. B_1_ is induced by inflammation and injury while B_2_ is constitutively expressed in vascular smooth muscle and endothelial cells. Via stimulation of phosphpolipase A_2_ and C, activation of endothelial B_2_ receptors by bradykinin promotes an enhanced cytosolic calcium concentration and the formation of prostaglandins and NO [20]. Our results show that B_2_ receptor blockade did not modify the vasorelaxant response induced by CNP in aortic rings from normotensive rats. Conversely, B_2_ receptor blockade enhanced the effect of CNP in SHR. It is known that BK induces COX-2 synthesis, which is related to synthesis of prostacyclin from endothelial cells [38], and given that SHR present high levels of BK [22] and an imbalance in the effect of prostanoids, this could explain our results with B_2_ receptor blocker inhibiting the increased vasoconstrictor effect of prostaglandins. In this regard, the possible crosstalk between both pathways, PG and CNP, in hypertensive states needs to be assessed.

Moreover, we demonstrated that, in the presence of an intact endothelium, CNP is able to induce relaxation in aorta from Wistar and SHR. However, CNP was substantially less potent in promoting aorta relaxation in hypertensive rats and this could be due to a reduced NO-dependent component in CNP induced relaxation, in association with lower increase of NOS activity in response to CNP in SHR. Removal of the aortic endothelium resulted in attenuation of CNP relaxant effect similar to that observed following NOS blockade in both groups, and the extent of NO-independent component was similar between normotensive and hypertensive rats. On the other hand, SNP-induced relaxation was different between groups, suggesting that a decrease in NO-mediated relaxation induced by CNP could be due to altered endothelium-dependent mechanism and/or vascular smooth muscle cells response in SHR. Several studies have suggested that reduced NO bioavailability in smooth muscle of SHR can be related to increased production of reactive oxygen species [13], but also vascular remodeling related to hypertensive states could be causing the lower response of vascular smooth muscle to a NO donor [24].

On the other hand, aortic endothelial NOS expression and basal NOS activity were higher in SHR than in Wistar rats, in agreement with our previous findings in 16 week old rats [6]. CNP induced an increase in NOS activity in intact endothelium aortic rings from both groups and in denuded aortas from Wistar rats. However, the present study demonstrated that CNP-induced NOS stimulation was lower in SHR than in Wistar rats. Previously, we demonstrated that CNP acute infusion stimulates NOS activity in both vascular endothelium and smooth muscle cells through NPR-C receptor activation in Wistar rats [7] and it was described that protein levels of NPR-C receptor are decreased in the aorta of SHR compared with normotensive rats [39] supporting our results. Considering that basal NOS activity is enhanced in SHR, we cannot dismiss the possibility that the lower vascular relaxation induced by CNP in these rats could also be related with the fact that this enzyme may be is in the upper limit of its response.

Interestingly, CNP is able to induce a similar decrease in the magnitude of peripheral vascular resistance in normotensive and hypertensive rats, but the blood flow response to this peptide differs between both groups of animals. The vasorelaxant effect of CNP could be mediated by NPR-C activation in normotensive rats, since changes in blood flow are similar between CNP and cANP_(4–23)_ infusions. In contrast, we showed that activation of NPR-C receptor promotes lower increase of blood flow than CNP infusion in hypertensive animals, suggesting the participation of other receptor in CNP-induced relaxation in SHR. Therefore, in a conductance vessels we demonstrated that CNP has a similar maximal vasodilator effect in normotensive and hypertensive rats, and that the vasorelaxant potency of NPR-C receptor activation is lower in SHR. Conversely, the vasodilator effect of CNP in peripheral resistance vessels is higher in hypertensive rats compared to normotensive rats. Moreover, activation of NPR-C pathway induces similar effect on blood flow compared to CNP administration in normotensive rats. Meanwhile, the vasodilator effect of NPR-C selective agonist is lower than CNP effect in SHR.

It is known that CNP can induce opening of potassium channels in different vascular beds and animal models. We tested involvement of potassium channels in CNP-induced relaxation in aortic smooth muscle cells from normotensive and hypertensive rats. In endothelium denuded aortic rings, the non-selective blockade of potassium channels with TEA blunted the vasorelaxant effect of CNP in aorta from both Wistar and SHR.

Our results suggest that aortic smooth muscle hyperpolarization elicited by CNP depends on inwardly rectifying K^+^ channels. Studies performed in isolated mesenteric artery support this finding: CNP mediates smooth muscle hyperpolarization and relaxation via NPR-C activation and the opening of Kir channels [40]. However, barium partially diminished the vasorelaxant response to CNP in aortic rings. This may be due to the fact that expression of the Kir channel is more abundant in the smooth muscle of autoregulatory vascular beds such as the coronary and cerebral circulations [41,42]. Indeed, the expression of the Kir channel appears to increase as the diameter of the artery decreases [43,44]. In addition, although Kir channels blockade diminished pEC_50_ in rings from hypertensive rats, no differences in pEC_50_ were observed in normotensive ones.

We also found that glibenclamide, a K_ATP_ channel blocker, induced no changes in the relaxant effect of CNP, indicating that ATP-dependent potassium channels are not involved in these mechanisms in Wistar and SHR.

On the other hand, functional BKCa channels are constitutively present in vascular smooth muscle cells from various species, while small-conductance Ca^2+^-activated K^+^ and intermediate-conductance Ca^2+^-activated K^+^ channels are more relevant in endothelial cells [45,46]. BKCa channels contribute to the control of vascular tone, promoting K^+^ outward current and leading to membrane hyperpolarization in vascular smooth muscle cells. They facilitate feedback regulation against the rise of intracellular Ca^2+^, membrane depolarization and vasoconstriction [47]. In smooth muscle cells of femoral arteries from SHR, Ca^2+^ influx and BKCa channel activity are increased [48], and the inhibition of BKCa channels by specific blockers causes strong constriction of the aorta [49]. Our results indicate that smooth muscle relaxation induced by CNP would be partially mediated by opening of BKCa channels given that IbTx partially decreased maximal response to CNP in Wistar rats. Although BKCa channel blockade also diminishes CNP-induced relaxation in hypertensive rats, we found that pEC_50_ was lower only in normotensive rats when endothelium-denuded rings were pre-incubated with IbTx.

Therefore, the results obtained with Kir and BKCa blockers in Wistar and SHR suggest that, although both potassium channels are involved in the vasodilator effect of CNP, this effect appears to be more sensitive to channel BKCa in Wistar rats, while CNP vasodilatory potency is more affected by opening of Kir channels in SHR.

In summary, we can conclude that CNP-induced relaxation in aorta involves NOS/sGC pathway activation in vascular endothelium. In normotensive and hypertensive rats, both NO production and hyperpolarization contribute to CNP dilator responses. The main finding of this study is that the vasodilator response to CNP in aorta is less potent in young adult male SHR than in normotensive rats, probably due to the impaired activity of the endothelial NO system in hypertensive rats. And although endothelium-independent response to CNP, mediated by opening of potassium channels, is similar in both SHR and Wistar rats, participation of the Kir and BKCa channels in vascular tone is different.

These results may be construed as an important step in the understanding of the possible cross-talk between CNP, NO and potassium channels in vascular function regulation under physiological and hypertensive states.

## Supporting Information

S1 Fig Contraction induced by phenylephrine in aortic rings from Wistar and SHR. PE: phenylephrine; Emax: maximal relaxant effect; pEC_50_: negative logarithm of the concentration of the agonist producing a half-maximal response. Phenylephrine response of aorta was expressed as % of KCl 90 mM response. Results are expressed as means ± SEM, n = 8 rats / group; ***p < 0.001 vs. Wistar Emax.
(TIF)

S2 Fig Relaxation induced by CNP in denuded aortic rings. Emax: maximal relaxant effect; pEC_50_: negative logarithm of the concentration of the agonist producing a half-maximal response. Results are expressed as means ± SEM, n = 8 rats / group. (A) Wistar concentration-response curves in the absence (ne) or presence (e) of an intact endothelium; ***p < 0.001 vs. Wistar e pEC_50_ values; ^##^p < 0.01 vs. Wistar e Emax values. (B) SHR concentration-response curves in the absence (ne) or presence (e) of an intact endothelium; ^††^p < 0.01 vs. SHR e pEC_50_ values; ^‡^p < 0.05 vs. SHR e Emax values.
(TIF)
