# Supplementary figures and images for: Vascular Tone Regulation Induced by C-Type Natriuretic Peptide: Differences in Endothelium-Dependent and -Independent Mechanisms Involved in Normotensive and Spontaneously Hypertensive Rats
Source: PLoS One. 2016 Dec 9;11(12):e0167817. doi: 10.1371/journal.pone.0167817 (PMC5147996; doi:10.1371/journal.pone.0167817)

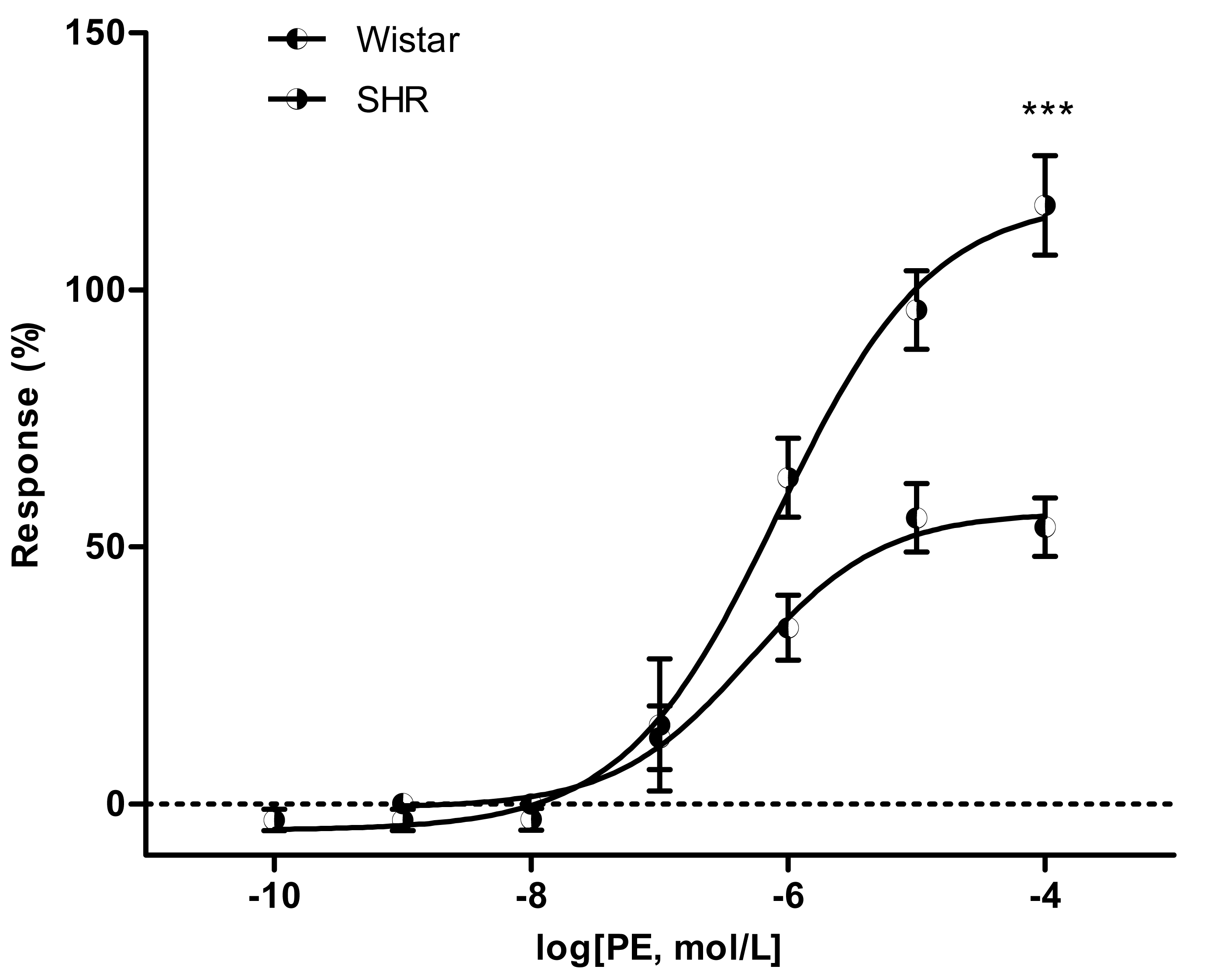

Supplement: S1 Fig — PE: phenylephrine; Emax: maximal relaxant effect; pEC50: negative logarithm of the concentration of the agonist producing a half-maximal response. Phenylephrine response of aorta was expressed as % of KCl 90 mM response. Results are expressed as means ± SEM, n = 8 rats / group; ***p < 0.001 vs. Wistar Emax. (TIF) [file pone.0167817.s001.tif]

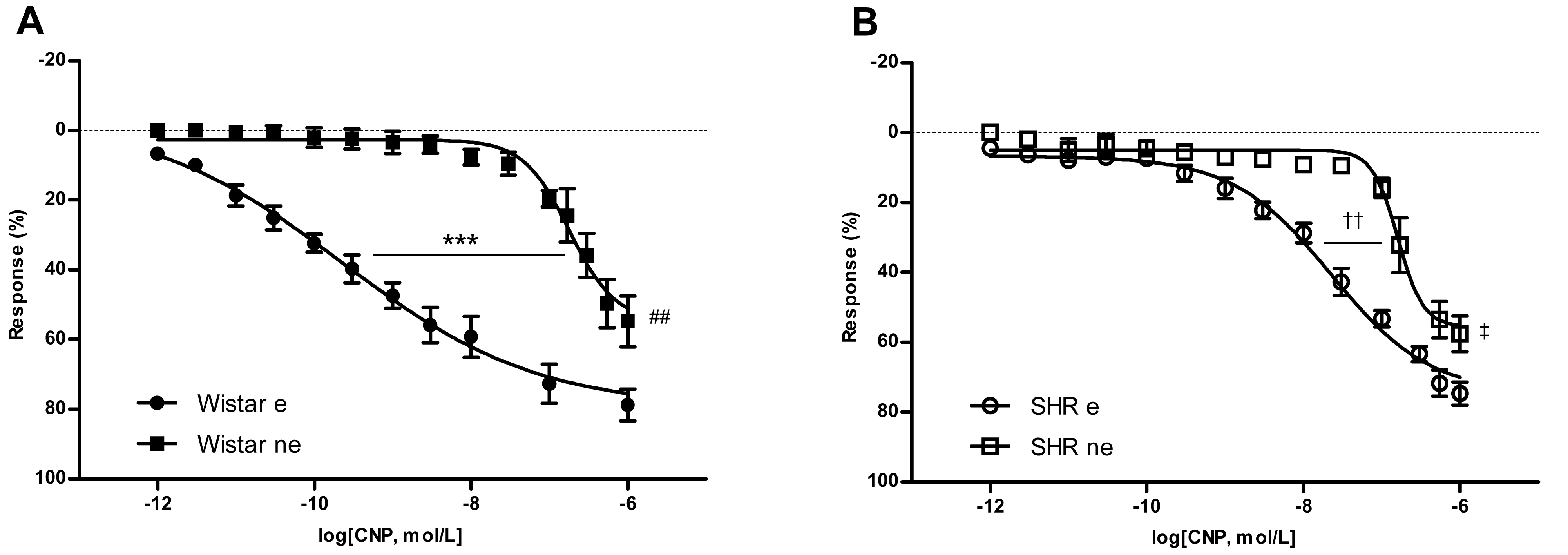

Supplement: S2 Fig — Emax: maximal relaxant effect; pEC50: negative logarithm of the concentration of the agonist producing a half-maximal response. Results are expressed as means ± SEM, n = 8 rats / group. (A) Wistar concentration-response curves in the absence (ne) or presence (e) of an intact endothelium; ***p < 0.001 vs. Wistar e pEC50 values; ##p < 0.01 vs. Wistar e Emax values. (B) SHR concentration-response curves in the absence (ne) or presence (e) of an intact endothelium; ††p < 0.01 vs. SHR e pEC50 values; ‡p < 0.05 vs. SHR e Emax values. (TIF) [file pone.0167817.s002.tif]
